# Machine-learning Approach for the Development of a Novel Predictive Model for the Diagnosis of Hepatocellular Carcinoma

**DOI:** 10.1038/s41598-019-44022-8

**Published:** 2019-05-30

**Authors:** Masaya Sato, Kentaro Morimoto, Shigeki Kajihara, Ryosuke Tateishi, Shuichiro Shiina, Kazuhiko Koike, Yutaka Yatomi

**Affiliations:** 1 Department of Clinical Laboratory Medicine, Graduate School of Medicine, The University of Tokyo, Tokyo, Japan; 2 Department of Gastroenterology, Graduate School of Medicine, The University of Tokyo, Tokyo, Japan; 3 Technical Research Laboratory, Shimadzu Corporation, Kyoto, Japan; 4 Department of Gastroenterology, Juntendo University, Tokyo, Japan

**Keywords:** Hepatocellular carcinoma, Cancer screening

## Abstract

Because of its multifactorial nature, predicting the presence of cancer using a single biomarker is difficult. We aimed to establish a novel machine-learning model for predicting hepatocellular carcinoma (HCC) using real-world data obtained during clinical practice. To establish a predictive model, we developed a machine-learning framework which developed optimized classifiers and their respective hyperparameter, depending on the nature of the data, using a grid-search method. We applied the current framework to 539 and 1043 patients with and without HCC to develop a predictive model for the diagnosis of HCC. Using the optimal hyperparameter, gradient boosting provided the highest predictive accuracy for the presence of HCC (87.34%) and produced an area under the curve (AUC) of 0.940. Using cut-offs of 200 ng/mL for AFP, 40 mAu/mL for DCP, and 15% for AFP-L3, the accuracies of AFP, DCP, and AFP-L3 for predicting HCC were 70.67% (AUC, 0.766), 74.91% (AUC, 0.644), and 71.05% (AUC, 0.683), respectively. A novel predictive model using a machine-learning approach reduced the misclassification rate by about half compared with a single tumor marker. The framework used in the current study can be applied to various kinds of data, thus potentially become a translational mechanism between academic research and clinical practice.

## Introduction

Hepatocellular carcinoma (HCC) is one of the commonest cancers and is the leading cause of cancer-related deaths worldwide^1^. Despite recent improvements in therapeutic interventions^2–5^, HCC is still associated with a poor prognosis in patients with an advanced disease stage^6^. Previous studies have reported the beneficial influence of regular surveillance for HCC in high-risk populations to detect HCC at an early stage^7–9^.

Regarding the suggested guidelines for HCC surveillance, whether tumor markers should be included in a surveillance program, along with ultrasonography, remains controversial, since the sensitivity or specificity of alpha-fetoprotein (AFP), which has been the most widely used tumor marker for HCC, by itself is unsatisfactory^10–13^. Because of the multifactorial nature of HCC^14^, it is difficult to predict its presence using a single biomarker. Therefore, combining multiple biomarkers to improve diagnostic accuracy is important. To date, other tumor markers, such as des-gamma-carboxyprothrombin (DCP)^15,16^ and the Lens culinaris agglutinin-reactive fraction of AFP (AFP-L3)^17,18^, have been proposed to complement the diagnostic accuracy of AFP. In addition to information on tumor markers, data on biomarkers of liver inflammation (aspartate aminotransferase [AST] and alanine aminotransferase [ALT]), fibrosis (platelet count)^19^, liver function (total bilirubin [TB] and albumin)^20^, and the hepatitis virus status are commonly available in daily clinical practice. These biomarkers alter the pretest probability for a diagnosis of HCC using tumor marker and thus are useful for predicting the presence of HCC.

Machine learning is a multidisciplinary field combining computer science and mathematics and focused on implementing computer algorithms capable of maximizing predictive accuracy from static or dynamic data sources using analytic or probabilistic models^21^. Combining clinical data using this analytical tool can enable the development of a novel model for HCC prediction. The aims of the present study are (1) to develop a machine-learning framework to establish the most appropriate model depending on the applied data, and (2) to apply this framework to existing data from HCC patients to develop an appropriate model for HCC prediction.

## Materials and Methods

### Patients

From all the patients who visited the liver clinic at the University of Tokyo Hospital between January 1997 and May 2016, we extracted 4242 patients (1311 HCC patients and 2931 non-HCC patients) for whom information on the presence (or absence) of HCC was available and who had undergone laboratory testing on at least one occasion. All the patients in the HCC-positive group had been diagnosed as having HCC at the time of their first visit and had received initial treatment at our institution. Patients who subsequently developed HCC during the follow-up period for chronic liver disease were included in the HCC-negative group in the current study. Patients for whom information on AFP, AFP-L3, DCP, AST, ALT, platelet count, alkaline phosphatase (ALP), gamma-glutamyl transferase (GGT), albumin, TB, age, sex, height, body weight, hepatitis B surface (HBs) antigen, and hepatitis C virus (HCV) antibody status were available were selected. Finally, we included 539 HCC patients and 1043 non-HCC patients with the required information in the current analysis.

The current study was performed in accordance with the ethical guidelines of the Declaration of Helsinki. This research project was approved by the ethics committee of the University of Tokyo (approval number, 11474). Informed consent was obtained in the form of an opt-out on the website. Patients who rejected participation in our study were excluded. The study design was also included in a comprehensive protocol for retrospective studies and was approved by the ethics committee of the University of Tokyo (approval number, 2058).

### Diagnosis of HCC

Hepatocellular carcinoma was diagnosed using dynamic computed tomography (CT) imaging, with hyper-attenuation during the arterial phase and washout during the late phase regarded as a definite sign of HCC^22^. When a definite diagnosis of HCC could not be made using CT, an ultrasound-guided tumor biopsy was performed and the pathological diagnosis was based on the Edmondson-Steiner criteria^23^.

### Development of graphical user interface machine-learning framework

**Figure 1:**
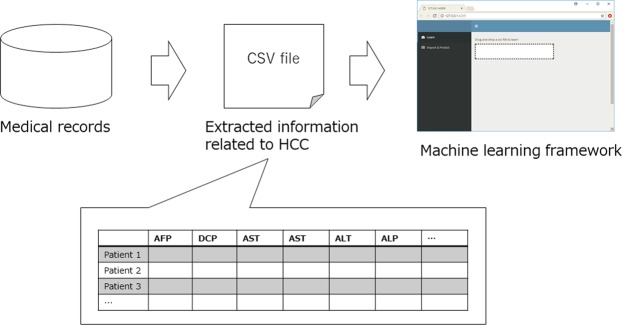
The concept of graphical user interface machine learning framework. Comma-separated values (CSV) dataset files with a labeled variable were dragged and dropped onto a dashboard, and the framework automatically implemented supervised learning and developed optimized classifiers and their respective hyperparameters.

To establish a predictive model, we developed a graphical user interface machine-learning framework using R version 3.4.3 (http://www.r-project.org) and the Shiny and Caret packages. The model had two main components. The first component consisted of the establishment of an algorithm. Comma-separated values (CSV) dataset files with a labeled variable were dragged and dropped onto a dashboard, and the framework automatically implemented supervised learning and developed optimized classifiers and their respective hyperparameters, depending on the nature of the data, using a grid-search method (Fig. 1). We used a linear logistic regression model for the linear classification. The Akaike information criterion was used for variable selection in this model. Algorithms including support vector machines using an RBF kernel, gradient boosting, random forests, neural networks, and deep learning were also used for a non-linear classification model. The classifiers and their respective hyperparameters are shown in Table 1. For deep learning model, we defined two dense layers using ReLU activation function with drop-out ratio of 0.5, and then added output layer with the sigmoid activation function. We compiled the model using binary cross entropy as the loss function. An RMS prop optimizer was used as a hyperparameter for the optimization of deep neural network. The framework automatically selected the best classifier and its respective hyperparameter for the prediction model based on a grid search. The detailed process of searching for the optimal hyperparameters was shown in Supplementary Table 1. Algorithm optimization (e.g., a heatmap of predictive accuracy in a support vector machine [SVM]) or materials to compare the accuracies among the classifiers (confusion matrix or receiver operating characteristic curve) were automatically created.

**Table 1:** Classifiers and their respective hyperparameters and R packages used.

Classifiers	Hyperparameters	R packages
Logistic regression model	—	stats
L1 penalized logistic regression model	lambda*	glmnet
L2 penalized logistic regression model	lambda*	glmnet
Elastic net penalized Logistic regression model	alpha^†^, lambda*	glmnet
RBF Support vector machine	C^‡^, sigma^§^	kernlab
Gradient Boosting	eta^||^, gamma^¶^, max_depth**, min_child_weight^††^, max_delta_step^‡‡^, subsample^§§^, colsample_bytree^||||^	xgboost
Random Forest	ntree^¶¶^, mtry***	randomForest
Neural Network	size^†††^, decay^‡‡‡^	nnet
Deep Learning ^||||||^	epochs^¶¶¶^, batch_size****, optimizer^††††^	keras tensorflow

^*^Scalar value, specifying the relative importance of the regularization function.

^†^An option to specify one or more values for the probability of a type-I error.

^‡^A parameter for the soft margin cost function, which specifies the allowance of a misclassification penalty for stability.

^§^A parameter to specify the complexity of the separation margin.

^||^A learning rate or step size shrinkage used in an update to prevent overfitting.

^¶^Minimal loss reduction required to make a further partition on a leaf node of the tree.

^**^Maximum depth of tree to control over-fitting; increasing this value makes the model more complex.

^††^Minimum sum of instance weight needed in a child node.

^‡‡^Maximum delta step allowed for each tree’s estimation.

^§§^Subsample ratio of training instance.

^||||^Subsample ratio of columns when constructing each tree.

^¶¶^Total number of trees included in the forest model.

^***^Number of features used in the construction of each tree.

^†††^Number of units in hidden layer (number of nodes in each hidden layer was set as 1).

^‡‡‡^A regularization parameter to avoid over-fitting.

^||||||^Fully connected neural network with 4 layers of neurons (16-64-64-2).

^¶¶¶^A single training iteration over the entire training data.

^****^Number of training samples processed at an iteration.

^††††^A device to adjust the deep learning model for optimal execution.

The second component consisted of the application of the developed model to a new dataset of interest. The CSV dataset of interest was dragged and dropped onto a dashboard, and the software applied the optimized classifiers and hyperparameters developed in the first component and outputted the probabilities of the respective labels.

### Statistical analysis

Continuous variables were expressed as the medians with the first and third quartiles, while categorical variables were expressed as frequencies (%). Comparisons were performed using the Wilcoxon rank-sum or chi-square test for quantitative and categorical variables, respectively. We adopted the approaches used in the developed framework described above to predict the presence of HCC. To evaluate the accuracy of the model, we randomly split a total of 1582 patients into three parts: (i) the training set (80%), which was used to build the model, (ii) the development set, which was used for tuning the model parameters, and (iii) the test set, which was used to evaluate the performance of each classifier and assessed the predictive accuracy of the developed model. We then used a receiver-operation characteristics (ROC) curve analysis to assess the predictive accuracy of our classifier. The area under the curve (AUC) was evaluated as the ability to predict the presence of HCC. The variable importance for class discrimination in the predictive model was assessed using the mean decrease in the Gini impurity^24^.

## Results

### Patient characteristics

Finally, we extracted 1582 patients from our database (539 HCC and 1043 non-HCC patients). The dataset did not contain any missing data. The patient characteristics are shown in Table 2. The proportions of patients with a male sex, HCV antibody-positivity, and HBs antigen-negativity were significantly higher among the HCC patients, compared with the non-HCC patients. The serum levels of AFP, AFP-L3, DCP, AST, ALP, GGT, and TB, and the patient age were also significantly higher among the HCC patients, whereas the serum ALT level, platelet count, and albumin level were lower.

**Table 2:** Patient characteristics (n = 1582).

Parameters	HCC patients (n = 539)	non-HCC patients (n = 1043)	*P* value
Sex, n (%)			<0.001
Female	167 (31.0)	483 (46.3)	
Male	372 (69.0)	560 (53.7)	
Age (years)	68 (63–74)	57 (48–66)	<0.001
HCV antibody			<0.001
Positive	382 (71.0)	630 (60.4)	
Negative	157 (29.0)	413 (39.6)	
HBs antigen			<0.001
Positive	78 (14.5)	254 (24.4)	
Negative	461 (85.5)	789 (75.6)	
AFP (ng/mL)	21 (7.8–91)	5.0 (3.0–10)	<0.001
AFP-L3 (%)	0.5 (0.0–92)	0.0 (0.0–0.5)	<0.001
DCP (mAU/mL)	24 (16–74)	16 (12–20)	<0.001
AST (U/L)	53 (38–77)	47 (29–77)	<0.001
ALT (U/L)	47 (29–74)	56 (30–95)	<0.001
Platelet Count (×10^4^/μL)	11.0 (7.9-15.7)	16.8 (12.0-22.1)	<0.001
GGT (IU/L)	55 (36–97)	49 (25–94)	<0.001
ALP (IU/L)	251 (193–323)	195 (155–250)	<0.001
Albumin (g/dL)	3.6 (3.2–4.0)	4.1 (3.8–4.3)	<0.001
TB (mg/dL)	0.8 (0.6–1.1)	0.8 (0.6–1.0)	<0.001
Height (cm)	161 (154–167)	162 (155–168)	0.11
Body weight (kg)	60.7 (53.0–68.0)	60.0 (52.0–68.0)	0.34

*Data were expressed as the median values (1^st^–3^rd^ quartiles).

### Predictive accuracy for HCC of each classifier

**Figure 2:**
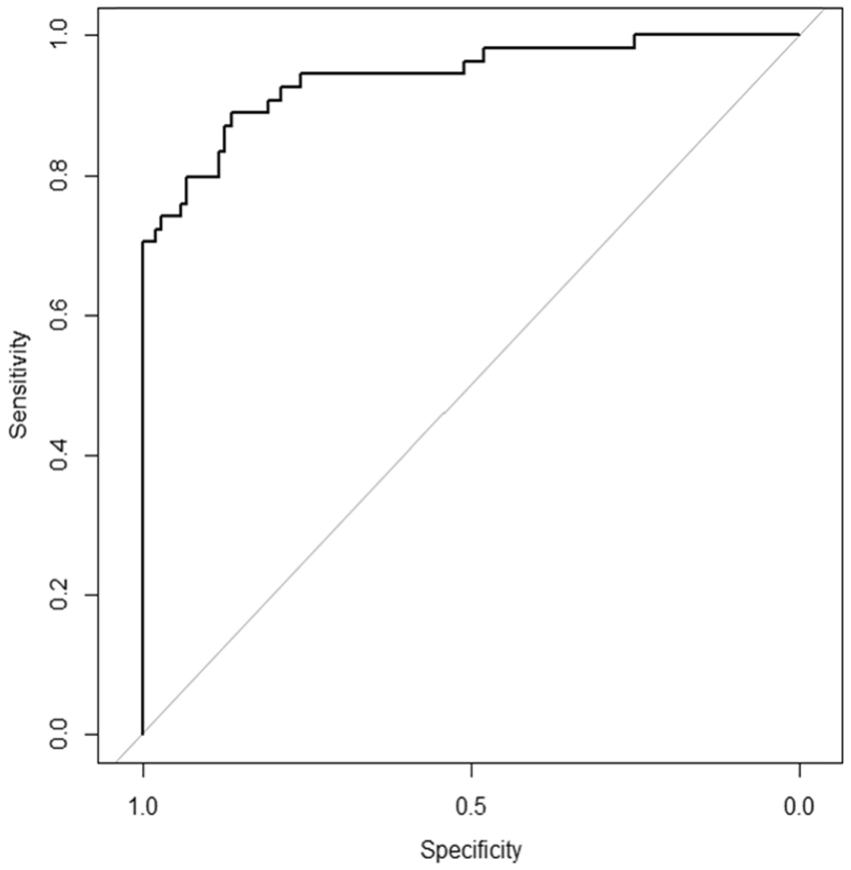
Receiver-operating characteristic curve for predicting the presence of HCC based on the optimal predictive model developed by our framework. The area under the curve for the prediction of HCC was 0.943.

Table 3 shows the predictive accuracy for HCC presence for each classifier using the optimum hyperparameter that provided the highest predictive value in each procedure. We assessed the predictive accuracy of the developed model in the test set. The predictive accuracy for HCC presence provided by gradient boosting was 87.34%, which was the highest among all the classifiers in our framework. The optimal hyperparameters of this classifier for the data used in the present study were eta = 0.08, gamma = 0.02, max depth = 1, min_child_ weight = 1.5, nround = 300, subsample = 0.5, and colsample_bytree = 0.9. An ROC analysis showed that the AUC, sensitivity, and specificity for this optimal classifier were 0.940, 93.27%, and 75.93%, respectively (Fig. 2). Deep learning was not an optimal classifier for the current data.

**Table 3:** Predictive accuracy for HCC presence of each classifier.

Classifier	Accuracy* (%)	Area under the curve
Logistic regression model	79.74	0.866
L1 penalized logistic bregression model	80.38	0.867
L2 penalized logistic regression model	81.64	0.884
Elastic net penalized logistic Regression model	80.38	0.884
Support vector machine (RBF kernel)	81.65	0.870
Gradient boosting	87.34	0.940
Random forest	86.08	0.923
Neural network	84.18	0.908
Deep learning	83.54	0.884

*A training/development/test split was used to evaluate the model.

### Assessment of variable importance for class discrimination of the predictive model

**Figure 3:**
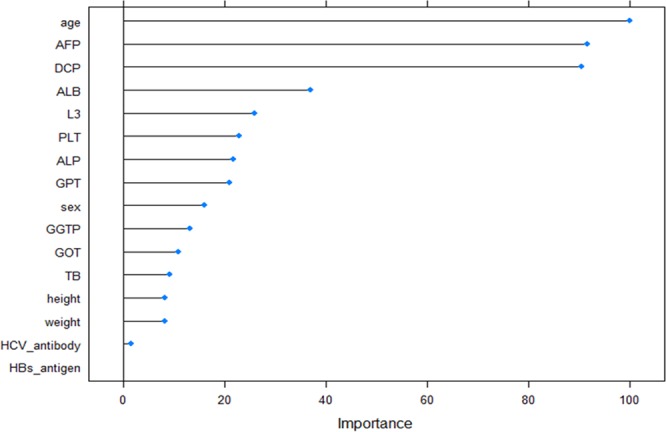
Mean decrease in the Gini impurity of the attributes as assigned using the optimized model. Patient age followed by three tumor markers were the most important variables for HCC prediction.

We then investigated the variable importance of the optimal predictive model using the gradient boosting developed in the current study. Figure 3 shows the mean decrease in the Gini impurity of this model. Patient age followed by three tumor markers and albumin level were the most important variables for HCC prediction.

### Predictive accuracy for HCC of single tumor markers

We also investigated the diagnostic accuracy of models using a single tumor marker. Using cut-offs of 200 ng/mL for AFP, 40 mAu/mL for DCP, and 15% for AFP-L3^25^, the accuracies of AFP, DCP, and AFP-L3 for HCC presence were 70.67%, 74.91%, and 71.05%, respectively. We also plotted the ROC curves for the prediction of HCC for three tumor markers (Supplementary Fig. 1). The AUCs for the prediction of HCC for AFP, DCP, and AFP-L3 were 0.766, 0.644, and 0.683, respectively.

## Discussion

In addition to tumor marker levels, biomarkers of liver inflammation, liver fibrosis, liver function, and the hepatitis virus status are commonly measured in daily clinical practice. These biomarkers can be used to predict the presence of HCC. Ideally, all clinically available information should be used for such predictions. In the current study, we developed a graphical user interface framework to establish the most appropriate model automatically depending on the applied data using a machine-learning approach and then assessed the accuracy of the model.

Model fitting is important for a successful predictive method. If the data is linearly separable, a linear model will fit the data^26,27^. However, if the data is linearly inseparable, a non-linear model will fit the data better. Therefore, classifiers should be selected depending on the nature of the data. Also, the learning parameters of each classifier should be tuned properly using a grid search method^28,29^ to obtain the ideal hyperparameters providing the highest predictive values. Using the optimal hyperparameter, gradient boosting (non-linear model) provided the highest accuracy (87.34%) for the data used in the current study. This model reduced the misclassification rate by about half, compared with a single tumor marker.

Personalization is one of the ultimate goals of modern medicine^30^. Predictive models provide a personalized assessment of the probability of a clinical event using patient-specific characteristics and have increasingly been incorporated into practice in the field of cancer medicine^31–33^. The framework developed in the current study can be used to identify optimal classifiers easily and can be applied to new datasets of interest containing various kinds of data, thus potentially becoming a translational mechanism between academic research and clinical practice.

Deep learning has enabled major breakthroughs in the processing of images, video, speech, and audio^34^. However, deep learning was not the optimal classifier in the current study. Deep learning requires a large polynomial sample size in terms of the dimensions of the input and an exponential sample in terms of the depth of the network to obtain ideal convergence boundaries^35^, which may be unrealistic requirements in clinical settings for ethical or methodologic reasons. Instead, identifying optimal classifiers and hyperparameters depending on the available data is important. The framework developed in the current study may help to provide optimal models.

The previous studies which compared the predictive performance of tabular data also showed the highest predictive performance of gradient boosting in the medical fields (e.g, urinary tract infections^36^, hip fractures^37^, sepsis^38^, or bioactive molecules^39^). Notably, Chiew *et al*. showed outstanding performance of gradient boosting compared to other machine learning algorithms for the risk prediction of suspected sepsis patients in the emergency department using relatively small number of sample^38^. Gradient boosting may be the best algorithm for the analysis of tabular data especially in the medical field where it is difficult to collect a large amount of data for ethical or methodologic reasons.

In the future, predictive models using machine learning approach may be implemented in electronic medical record system and may offer decision support to improve patient outcomes and reduce clinical diagnosis error in daily medical practice. The accuracy of diagnostic algorithm based on machine learning approach depends on the number of samples for training^40^. Larger quantities of multidimensional medical data will be stored in the future, potentially improve the accuracy of machine learning based classifier. Discrepancy of disease distribution between train and test samples is also an important factor for the performance of each classifier. The predictive model developed in the current study is based on the data of tertiary referral center requires. Therefore, further study with external validation in a community-and clinic based population is needed to assess the practical performance of the current model.

In conclusion, the framework developed in the current study provided a novel predictive model of HCC, producing an area under the curve of 0.943. This model reduced the misclassification rate by about half, compared with that for a single tumor marker. The current framework can be applied to various kinds of data, and thus could potentially become a translational mechanism between academic research and clinical practice.

## Supplementary information

Supplementary Information

## Data Availability

The datasets generated during the current study are available from the corresponding author on reasonable request.
