# Supplementary material for: Machine-learning Approach for the Development of a Novel Predictive Model for the Diagnosis of Hepatocellular Carcinoma
Source: Sci Rep. 2019 May 30;9:7704. doi: 10.1038/s41598-019-44022-8 (PMC6543030; doi:10.1038/s41598-019-44022-8)

Original Article: Liver, Pancreas, and Biliary Tract

Machine-learning Approach for the Development of a Novel Predictive Model for the Diagnosis of Hepatocellular Carcinoma

Masaya Sato[1,2], Kentaro Morimoto[3], Shigeki Kajihara[3], Ryosuke Tateishi[2], Shuichiro Shiina[4], Kazuhiko Koike[2], Yutaka Yatomi[1]

[1]Department of Clinical Laboratory Medicine, Graduate School of Medicine, The University of Tokyo, Tokyo, Japan; [2] Department of Gastroenterology, Graduate School of Medicine, The University of Tokyo, Tokyo, Japan; [3]Technical Research Laboratory, Shimadzu Corporation, Kyoto, Japan; [4]Department of Gastroenterology, Juntendo University, Tokyo, Japan

Running Head

Development of a HCC predictive model

Corresponding author:

Masaya Sato, M.D., Ph.D., Department of Clinical Laboratory Medicine, Graduate School of Medicine, The University of Tokyo, 7-3-1 Hongo, Bunkyo-ku, Tokyo 113-8655, Japan

Tel: +81-3-3815-5411

Fax: +81-3-5689-0495

E-mail: masayasato0407@gmail.com

Word count (text): 2043 words

Tables: 3

Figures: 3

Competing Interest Statement

None of the authors have any competing interest.

Author Contributorship statement

The seven authors are justifiably credited with authorship, according to the authorship criteria.

In detail: MS – conception, design, analysis and interpretation of data, drafting of the manuscript, final approval given; KM－analysis and interpretation of data, final approval given; SK－analysis and interpretation of data, final approval given; RT – analysis and interpretation of data, critical revision of manuscript, final approval given; SS – collection and assembly of data, final approval given; KK – critical revision of manuscript, final approval given; YY– critical revision of manuscript, final approval given

Acknowledgement

This research was supported by the AMED under Grant Number JP17fk0210304 and JP18fk0210040.

The English in this document has been checked by professional editor, native speakers of English.

Data Availability Statement

The datasets generated during the current study are available from the corresponding author on reasonable request.

Supplementary Table 1. The detailed process of searching for the optimal hyperparameters

Classifiers	Details of hyperparameter searching
L1 penalized logistic regression model	lambda was searched from 1e-01 to 1e-10<br>(1e-01, 1e-02, 1e-03, 1e-04, 1e-05, 1e-06, 1e-07, 1e-08, 1e-09, 1e-10)
L2 penalized logistic regression model	lambda was searched from 1e-01 to 1e-10<br>(1e-01, 1e-02, 1e-03, 1e-04, 1e-05, 1e-06, 1e-07, 1e-08, 1e-09, 1e-10)
Elastic net penalized Logistic regression model	alpha was searched from 0.1 to 0.9 (at intervals of 0.1)<br>lambda was searched from 1e-01 to 1e-10<br>(1e-01, 1e-02, 1e-03, 1e-04, 1e-05, 1e-06, 1e-07, 1e-08, 1e-09, 1e-10)
RBF Support vector machine	C was searched from 1.0 to 5.0 (at intervals of 0.1)<br>sigma was searched from 0.001 to 0.1 (at intervals of 0.001)
Gradient Boosting	1st step<br>max_depth was searched from 1.0 to 5.0 (at intervals of 1.0)<br>min_child was searched from 0.001 to 0.1 (at intervals of 0.001)<br>nround was searched from 200, 300, 500, and 1000<br><br>2nd step<br>Using the optimal hyperparameters derived in the 1st step<br>colsample_bytree was searched from 0.5 to 1.0 (at intervals of 0.1)<br>subsample was searched from 0.5 to 1.0 (at intervals of 0.1)<br><br>3rd step<br>Using the optimal hyperparameters derived in the 1st and 2nd steps,<br>eta was searched from 0.01 to 0.15 (at intervals of 0.01)<br><br>4th step<br>Using the optimal hyperparameters derived in the 1st, 2nd, and 3rd steps,<br>Gamma was searched from 0.0 to 0.15 (at intervals of 0.01)
Random Forest	mtry was searched from 1.0 to 10.0 (at intervals of 1.0)<br>ntree was searched from 100, 200, 300, 500, and 1000
Neural Network	size was searched from 1.0 to 10.0 (at intervals of 1.0)<br>decay was searched from 0.1 to 1.0 (at intervals of 0.1)
Deep Learning	epochs was searched from 10 to 100 (at intervals of 10)<br>batch_size was searched from 10 to 30 (at intervals of 10)

Figure legend

Supplementary Figure 1. Receiver-operating characteristics curve for predicting the presence of HCC presence based on single tumor markers. (a) AFP: area under the curve for HCC prediction, 0.766. (b) DCP:-area under the curve for HCC prediction, 0.644. (c) AFP-L3: area under the curve for HCC prediction was 0.683.

Supplementary Figure 1a

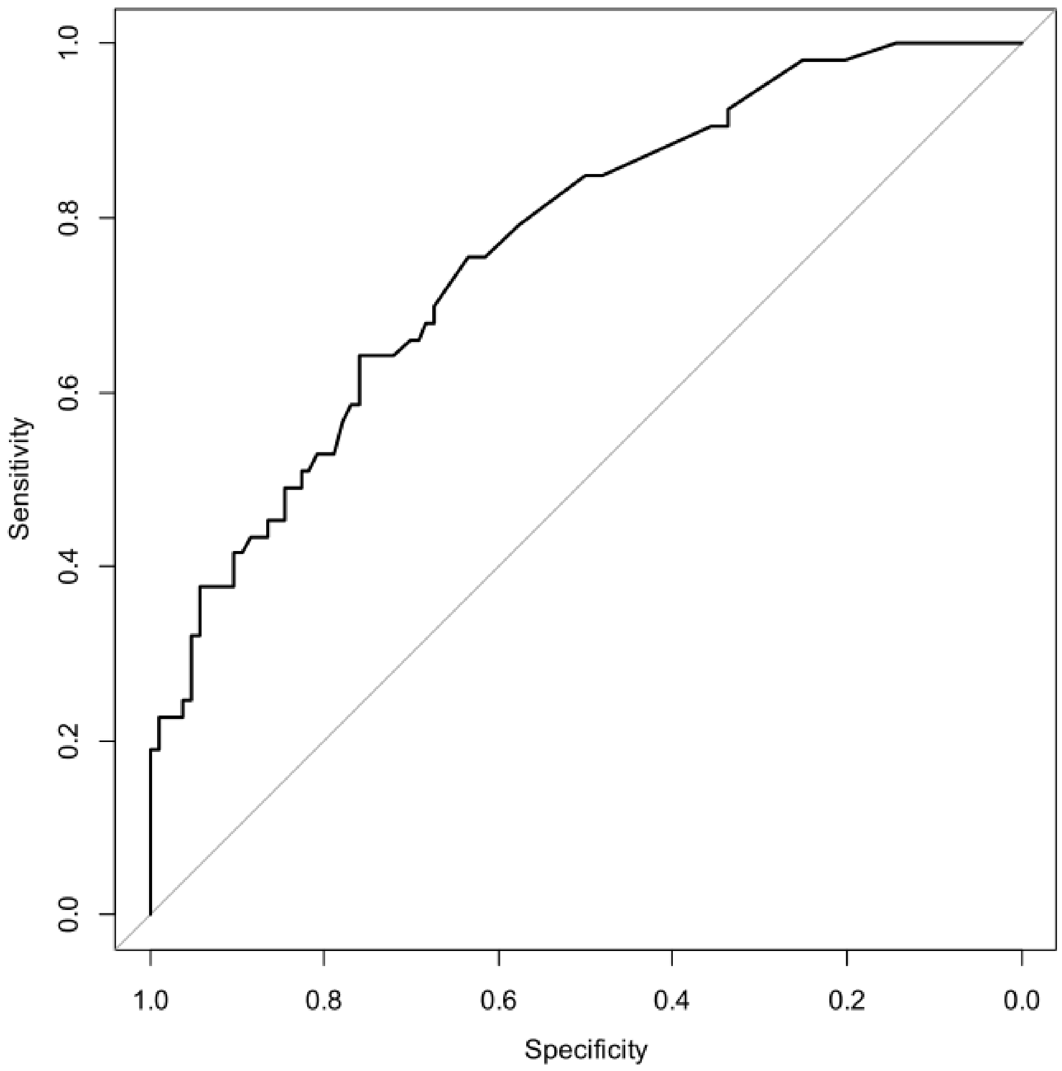

Supplementary Figure 1b

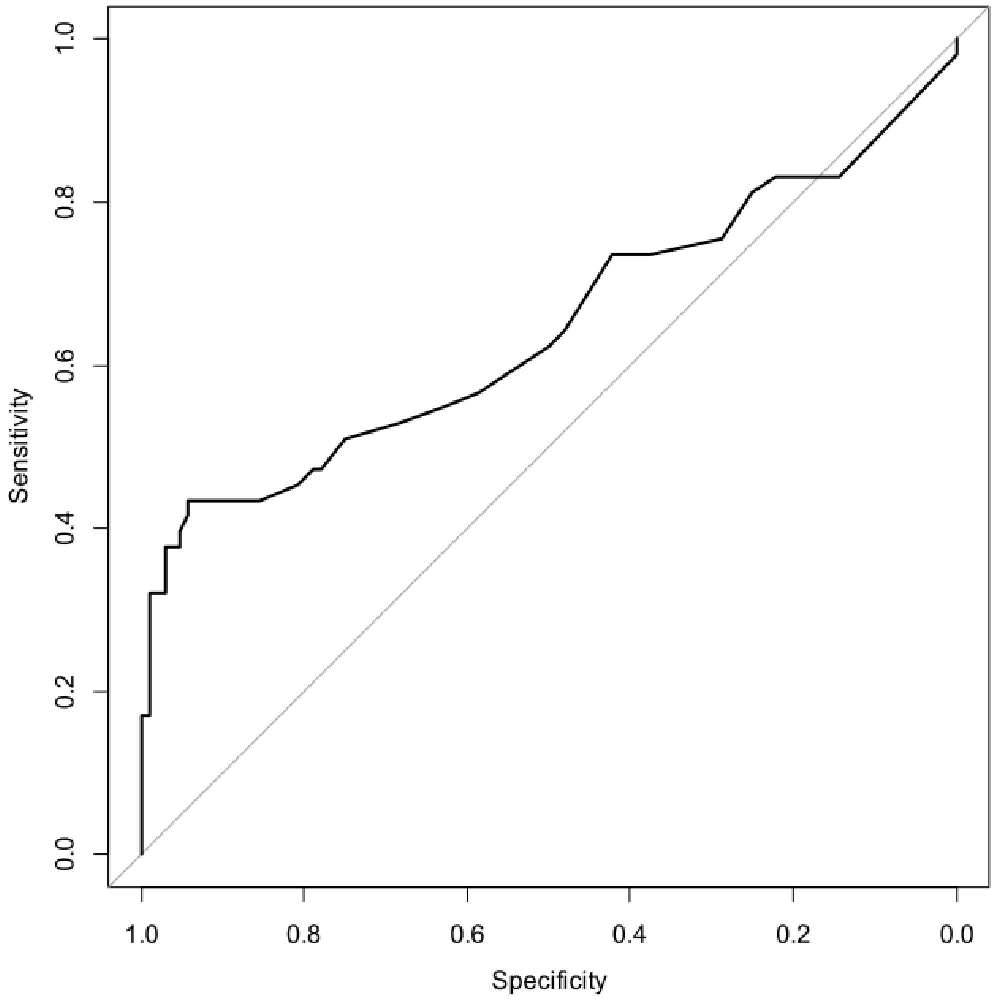

Supplementary Figure 1c

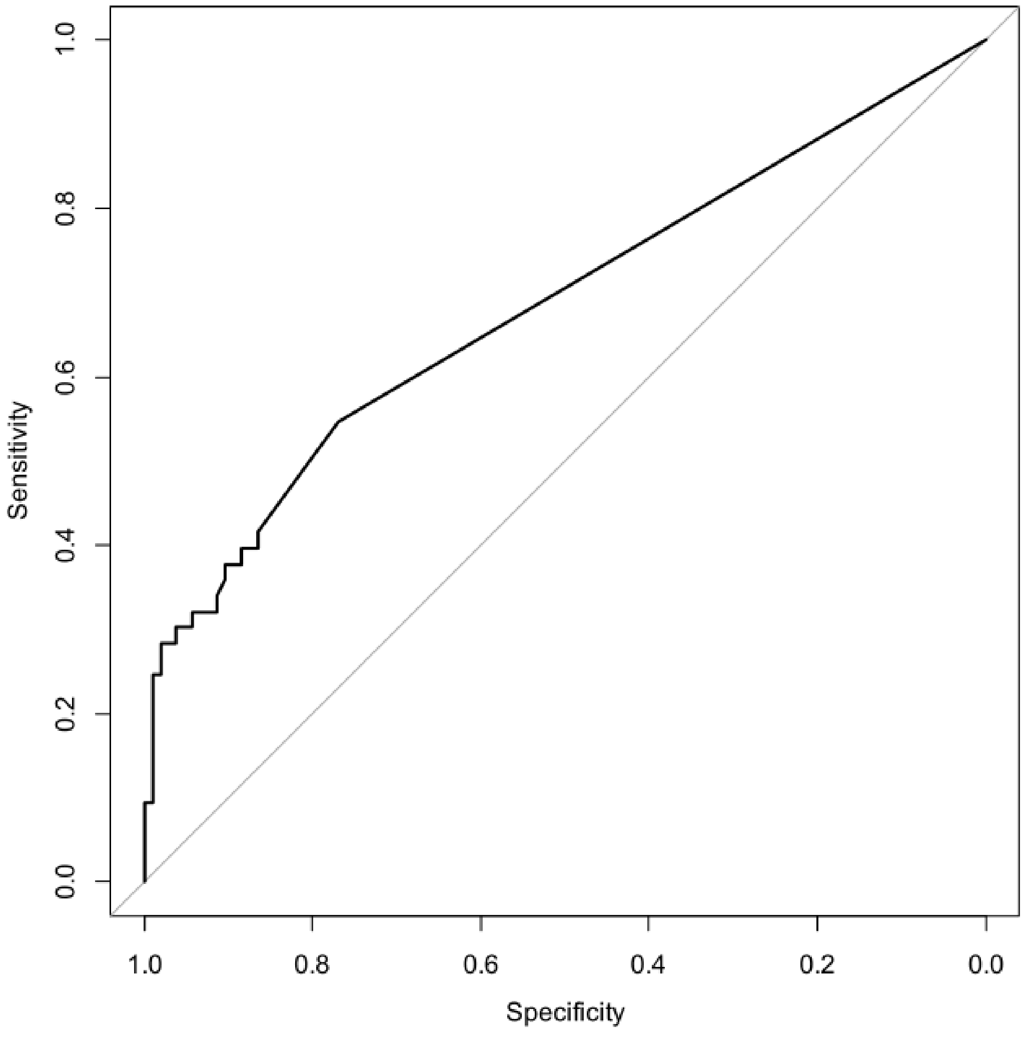

Supplement: Supplementary file 1 — Supplementary Information [file 41598_2019_44022_MOESM1_ESM.pdf]
